# The Relationship Between Big Five Personality Traits and Psychotic Experience in a Large Non-clinical Youth Sample: The Mediating Role of Emotion Regulation

**DOI:** 10.3389/fpsyt.2018.00648

**Published:** 2018-12-04

**Authors:** Jingyu Shi, Yuhong Yao, Chenyu Zhan, Ziyu Mao, Fang Yin, Xudong Zhao

**Affiliations:** ^1^Shanghai East Hospital, Tongji University School of Medicine, Shanghai, China; ^2^Division of Medical Humanities & Behavioral Sciences, Tongji University School of Medicine, Shanghai, China; ^3^Students Counseling Center, Tongji University, Shanghai, China

**Keywords:** emotion regulation, psychotic experience, prodromal symptoms, personality traits, youth

## Abstract

**Objective:** Despite a long history of interest in personality traits and psychosis, the association between personality traits and psychotic experiences in the general population is not yet well understood. One possible factor that could influence the degree of distress from psychotic experiences is emotion regulation. The purpose of this study was to explore whether the association between personality and psychotic symptoms is already apparent in non-clinical youth as well as the mediating role of emotion regulation strategies between personality traits and psychotic experiences.

**Methods:** Three thousand one hundred and forty seven college students were surveyed via self-report questionnaires measuring the Five-Factor model of personality, emotion regulation strategies, and psychotic experiences.

**Results:** Neuroticism was found to be significantly positively correlated with psychotic experiences, while Extraversion, Openness, Agreeableness, and Conscientiousness were found to be significantly negatively correlated. Both the suppression and reappraisal strategies mediated the relationship between personality traits and psychotic experiences.

**Conclusion:** Our findings suggest that youth with certain personality traits are more likely to have psychotic experiences. The reappraisal emotion regulation strategy could serve as a protective factor against the distress of psychotic experiences.

## Introduction

Attention has long been paid to exploring the role of personality in psychopathology. The Five Factor Model (FFM) of Personality, known as the “Big Five” model, is the most well-known model of personality (1). The five personality traits of the FFM are neuroticism, extraversion, openness, agreeableness, and conscientiousness. Neuroticism refers to the vulnerability to emotional instability and self-consciousness. Openness is characterized by the cognitive disposition to creativity and esthetics. Agreeableness and extraversion focus on the interpersonal relationship: Extraversion reflects the tendency to be gregarious, enthusiastic, assertive, and to seek excitement, whereas agreeableness refers to the tendency to be warm, kind, gentle, trusting, and reliable. Conscientiousness is understood as the tendency toward dutifulness and competence. These five personal traits are viewed as the most basic dimensions of personality (2). People with schizophrenia were found to demonstrate higher levels of Neuroticism. This construct of personal traits has a strong predictive effect on the level of positive symptoms in psychosis studies (3). FFM traits may promote a vulnerability to developing psychosis disorders. High levels of neuroticism prior to onset reflects a vulnerability to feeling anxious and being distressed. This trait was found to be a risk factor for the development of schizophrenia (4–6), while a high level of extraversion could reduce the risk (6). There is also assertive evidence for the link between the FFM dimensions extraversion, openness and agreeableness, and schizophrenia. In samples with first-episode schizophrenia and chronic psychosis, lower levels of both extraversion and agreeableness have been associated with higher levels of positive psychotic symptoms and relapse (7).

College students are in the transitional period from adolescence to young adulthood. It is particularly interesting to explore the personality–psychopathology relationships in this population, because it demonstrates a period that is linked to neurological (8) and psychosocial developmental changes that potentially influence an individual's mental health and personality (9). College students belong to a population with a high incidence of psychosis. However, little is known about the relationship between personality traits and psychotic experiences among college students. Psychotic experiences are relatively common in late adolescence and young adulthood, and co-present with other psychiatric problems (10). Psychotic experiences may potentially be related to an increased risk for the onset of psychotic and other psychiatric disorders later in life, especially if the psychotic experiences affect social functioning and last over time (11, 12). Therefore, it was relevant to investigate how personality traits are associated with psychotic experiences in youth.

One way to address this question is to explore how different personality traits associate with emotion regulation (ER) styles, such as suppression vs. cognitive reappraisal, and furthermore relating to the distress of psychotic experiences. Emotion regulation can be understood as the conscious and unconscious processes by which one's emotional experience and expression are influenced (13). The studies of Gross and John (14) are focused on two different main strategies: cognitive reappraisal, which refers to altering the way of thinking about the event (an adaptive strategy) and expressive suppression, which means managing the expressive behavioral response to the event (a maladaptive strategy). It has been found that different personality traits lead to distinct ER styles: Gresham and Gullone (15) reported that higher levels of Extraversion and Openness could predict higher use frequency of cognitive reappraisal, while higher levels of neuroticism and lower levels of extraversion predicted a higher frequency of using suppression. Individuals with schizophrenia tend to use maladaptive emotion regulation strategies such as suppression and are less likely to use adaptive emotion regulation strategies [e.g., reappraisal; (16)]. For instance, compared to healthy controls, individuals with schizophrenia and those at clinical high risk for psychosis (CHR) report more frequent use of suppression (17), which has been further linked to more severe auditory hallucinations and greater impairments in social functioning (18–20). In addition, individuals with schizophrenia have less chance than healthy controls to engage in emotional acceptance (16, 21, 22). There is also evidence that individuals with schizophrenia and those at CHR are less likely to employ reappraisal [i.e., changing one's understanding about a situation to alter its emotional impact; (17)] than controls (18, 20, 23, 24). Likewise, some studies in general population have indicated that lower possibility of utilization of adaptive strategies (25) or the use of maladaptive emotion regulation strategies (26) was significantly associated with greater severity of delusions. Besides, Henry et al. (27) found that in a sample of the general population, psychometric schizotypy was significantly linked to the utilization of expressive suppression. The findings of these previous studies suggest that positive psychotic symptoms could be related to a tendency toward more usage of maladaptive emotion regulation strategies.

Although these previous findings provide some evidence for the role of personality traits and emotion regulation in psychotic experience, several unanswered questions remain. There is currently no research focusing on the relationship between personality traits, emotion regulation and psychotic experience in non-clinical youth. Therefore, the aim of the current study is to investigate (1) the relationship between Big Five personality traits and psychotic experiences in a non-clinical youth population and (2) the mediating role of emotion regulation between personality traits and psychotic experiences. Based on earlier work, we hypothesized that:

1. Neuroticism is positively correlated with psychotic experiences and other dimensions of personality traits are negatively correlated.
2. Both of the strategies of emotion regulation mediated the relationship between personality traits and psychotic experiences.

## Methods

### Participants

A sample of 3,147 college freshmen were recruited at a Chinese university. Among them 3,120 students completed the questionnaires. The effective response rate was 99.14%. The participants' ages ranged from 17 to 25 years, with an average of 19.20 years (SD = 0.708). There were 1,224 female students (39.2 %) and 1,896 male students (60.8%). Prior to the study all the participants signed written informed consent. On behalf of the participants under 18 years the written informed consent from the caretakers was also obtained.

### Measures

#### Psychotic Experience

The 16-item version of Prodromal Questionnaire (PQ-16) measures psychotic experience. The PQ-16 is a shortened version of the 92-version of the PQ (28), and it has been validated in a non-psychotic help-seeking population (29) and in a sample of Chinese college students (30, 31). It consists of nine items referring to hallucinations and perceptual abnormalities, five items referring to delusional thoughts, paranoia and unusual thinking content, and the other two items concerning negative symptoms (28). According to the subjective psychotic experiences during the last month, the items were marked as yes or no. If an answer for an item was marked as “no,” this item was rated 0; when “yes” was selected for an item, the severity of the distress from the psychotic experience ranged from 0 to 3 (possible responses: 0 no distress, 1 mild distress, 2 moderate distress, and 3 severe). In the current study, the internal consistency reliability of PQ-16 was good (Cronbach's α = 0.78).

#### Emotion Regulation Strategies

The emotion regulation questionnaire [ERQ, (14)] was applied to measure the habitual use of two emotion regulation strategies (reappraisal and suppression). Reappraisal refers to acts of changing one's cognitive meaning to reduce emotion. In contrast, suppression is a way of managing response by inhibiting any ongoing expressive behavior. The ERQ is a 10-item self-report instrument with a 7-point Likert scale (1 = strongly disagree, 7 = strongly agree). Sample items for reappraisal include “I control my emotions by changing the way I think about the situation I am in,” and items for suppression include “I control my emotions by not expressing them.” A higher score suggests a higher frequency of using one specific strategy. The validity and reliability of the Chinese ERQ were satisfactory according to Wang et al. (32). In this study, the Cronbach's α of the ERQ reappraisal and suppression subscale was 0.783 and 0.772 respectively.

#### The Big Five Personality Traits

The Big Five personality factors were measured using Wang et al.'s (33) 40-item Chinese Big Five Personality Inventory Brief Version (CBF-PI-B), which was developed based on the Big Five personality traits model (34). The scale includes 5 traits, namely openness, conscientiousness, extraversion, agreeableness, and neuroticism. Each dimension is assessed with 8 items. It is rated on a five-point Likert scale from 1 (very inaccurate) to 5 (very accurate). Sample items include, “I often feel scared,” “I work or study hard,” “My imagination is quite rich,” “I like to take risks,” and “I often worry about something unimportant.” The scores for each dimension range from 8 to 40. CBF-PI-B was tested to have good psychometric properties. Internal consistency reliabilities (Cronbach's α) of the CBF-PI-B ranged from 0.764 to 0.814; the test-retest reliabilities ranged from 0.672 to 0.811 (33).

### Procedure

All the participants volunteered to take part in this study, which took place at the end of the mental health education course. After they signed the informed consent form, participants completed a questionnaire survey, including measures from the Prodromal Questionnaire, the Emotion Regulation Questionnaire, and the Big Five Personality Inventory in a quiet classroom environment. It took ~15 min for the participants to complete all the surveys.

### Data Analysis

The Statistical Package for the Social Sciences (SPSS 17.0) was used to conduct all statistical procedures. Pearson correlation was applied to examine how levels of psychotic experience and emotion regulation strategies as well as personality traits were related. In addition, Bonferroni corrections were applied to adjust for multiple comparisons; multiple regression analysis and the Sobel test were calculated to explore the mediating effect of emotion regulation on the relationship between personality traits and psychotic experiences. The Sobel test is a method of testing the significance of a mediation effect. The test is based on the work of Sobel (35). In mediation, the relationship between the independent variable and the dependent variable is hypothesized to be an indirect effect that exists due to the influence of a third variable (the mediator).

## Results

### Demographical and Descriptive Data

The 3,120 college students aged from 17 to 25 years, with an average of 19.20. Among the participants, 1,224 (39.2%) were females. Descriptive statistics for all questionnaire measures are presented in Table 1.

**Table 1:** Descriptive statistics (*N* = 3120).

	**Mean**	**SD**
Psychotic experience	1.31	2.775
Reappraisal	30.54	5.606
Suppression	16.24	4.631
Neuroticism	17.53	6.486
Extraversion	25.87	6.260
Openness	29.79	6.280
Conscientiousness	30.34	5.738
Agreeableness	31.70	5.051

### Correlations Between Personality Traits, Psychotic Experiences, and Emotion Regulation

After the Bonferroni correction, the correlation shows that there are significant associations between psychotic experience, the five personality traits and two emotion regulation strategies (*P* = 0.000; Bonferroni corrected *P* < 0.002). The correlations are presented in Table 2.

**Table 2:** Correlations between personality traits, psychotic experience and emotion regulation (*n* = 3120).

	**Neuroticism**	**Extraversion**	**Openness**	**Conscientiousness**	**Agreeableness**	**Reappraisal**	**Suppression**
Extraversion	−0.309**					
Openness	−0.236**	0.408**				
Conscientiousness	−0.294**	0.301**	0.346**			
Agreeableness	−0.243**	0.323**	0.325**	0.353**		
Reappraisal	−0.231**	0.205**	0.255**	0.314**	0.313**	
Suppression	0.317**	−0.261**	−0.089**	−0.067**	−0.197**	−0.029
Psychotic experience	0.521**	−0.237**	−0.125**	−0.190**	−0.167**	−0.140**	0.283**

** *P = 0.000*.

### The Mediating Role of Cognitive Reappraisal Strategy Between Personality Traits and Psychotic Experiences

According to Baron and Kenny (36), Sobel (35), Goodman (37), and MacKinnon et al. (38), multiple regression analysis was used to test whether emotion regulation mediated the relationship between personality traits and psychotic experience. The analysis was conducted separately for each emotion regulation strategy so that there are five regression analyses for the cognitive reappraisal strategy. At step 1, regression analysis was conducted with psychotic experience as the dependent variable and each of the five personality traits as independent variables. The results of the regression analysis indicated that all five personality factors had significant effect on psychotic experience (β = −0.069~0.514; *P* < 0.01); at step 2, regression analysis was conducted with the reappraisal strategy as the dependent variable and each of the five personality traits as independent variables. The results of the regression analysis indicated that all five personality factors had significant effect on the use frequency of the reappraisal strategy (β = −0.223~0.344; *P* < 0.01); at step 3, the Sobel test was conducted for the five models, and the results suggested that the cognitive reappraisal strategy significantly mediated the relationship between all of the five personality factors and psychotic experiences. Results of the Sobel test for the five models are presented in Table 3.

**Table 3:** Sobel test for the mediating role of reappraisal between personality traits and psychotic experiences.

	**a**	**b**	**s_a_**	**s_b_**	**Z**
**Model 1**					
Neuroticism—reappraisal—psychotic experiences	−0.192	−0.064	0.015	0.009	6.216***
**Model 2**					
Extraversion—reappraisal—psychotic experiences	0.211	−0.064	0.016	0.009	−6.259***
**Model 3**					
Openness—reappraisal—psychotic experiences	0.272	−0.064	0.015	0.009	−6.620***
**Model 4**					
Conscientiousness—reappraisal—psychotic experiences	0.336	−0.064	0.016	0.009	−6.735***
**Model 5**					
Agreeableness—reappraisal—psychotic experiences	0.369	−0.064	0.019	0.009	−6.678***

*** *P < 0.001*.

### The Mediating Role of the Expressive Suppression Strategy Between Personality Traits and Psychotic Experiences

There are five supposed models to test whether the emotional suppression strategy mediated the relationship between personality traits and psychotic experiences. We also applied regression analysis with three steps for each model. At step 1, regression analysis was conducted with psychotic experiences as the dependent variable and each of the five personality traits as independent variables. At step 2, regression analysis was conducted with the suppression strategy as the dependent variable and each of the five personality traits as independent variables. The results of the regression analysis indicated that except for Conscientiousness (β = −0.032; *P* > 0.05), the four other personality factors had significant effect on the use frequency of the suppression strategy (β = −0.059~*0.314*; *P* < 0.01); at step 3, the Sobel test was conducted for the five models, and the results suggested that the expressive suppression strategy significantly mediated the relationship between the four personality factors (except Conscientiousness) and psychotic experiences. Results of the Sobel tests for the five models are presented in Table 4.

**Table 4:** Sobel test for the mediating role of suppression between personality traits and psychotic experiences.

	**a**	**b**	**s_a_**	**s_b_**	**Z**
**Model 1**					
Neuroticism—suppression—psychotic experiences	0.224	0.139	0.012	0.010	11.149***
**Model 2**					
Extraversion—suppression—psychotic experiences	−0.199	0.139	0.013	0.010	−10.291***
**Model 3**					
Openness—suppression—psychotic experiences	−0.043	0.139	0.013	0.010	−3.218***
**Model 4**					
Conscientiousness—suppression—psychotic experiences	−0.026	0.139	0.014	0.010	−1.841
**Model 5**					
Agreeableness—suppression—psychotic experiences	−0.189	0.139	0.016	0.010	−9.001***

*** *P < 0.001*.

## Discussion

Personality traits and emotion regulation strategies are potential protective factors against the distress from psychotic experiences. This study was conducted in a large non-clinical sample of youth and provides some of the first evidence linking personality traits, emotion regulation and psychotic experiences. As hypothesized, neuroticism was found to be significantly positively correlated with psychotic experiences, while extraversion, openness, agreeableness and conscientiousness were found to be significantly negatively correlated. These findings are comparable with the conclusions of previous studies (39–42). High neuroticism and low extraversion, low agreeableness, low openness as well as low conscientiousness might be unfavorably related to psychotic experience. These FFM traits perhaps, in part, represent structural tendencies in affect, cognition, and behavior in individuals that might elicit higher levels of stress, contribute to social isolation and reduce opportunities for disconfirmation of psychotic interpretation. However, in the previous studies a positive association was found between openness and the frequency of psychotic symptoms; while in the current study openness was found to be negatively correlated with the distress of psychotic experiences. The potential reasons are as follows: First, individuals who exhibit openness may possess extensive and deep cognitive contents as well as genuine and complex life experiences. Their openness to emotional situations in general makes them optimistic and enables them to cope with negative emotion (43). In addition, the personality traits, such as openness, of psychotic patients change during the different stages of psychosis. Xu et al. (44) found that the level of openness in the patients was obviously higher than the level of openness before onset and while in remission, so the association between personality traits and psychotic experience for the clinical and non-clinical population could be different. Another potential explanation is that there are mediators of the relationship between the personality traits and psychotic symptoms, a finding that needs to be explored further.

In addition, we found that extraversion, openness, agreeableness, and conscientiousness are significantly positively related to the use frequency of the reappraisal strategy and negatively related to the use frequency of the suppression strategy; neuroticism is significantly negatively associated with the use frequency of the reappraisal strategy and positively related to the use frequency of the suppression strategy These results are consistent with previous studies (15, 45–50). This study indicates that individuals with high levels of neuroticism tend to be pessimistic in adopting any strategy for regulating their emotions. Other personality traits may enable individuals to make cognitive responses in the emotional regulation strategies.

The findings also suggest that the use frequency of the reappraisal strategy is significantly negatively correlated with psychotic experiences; the use frequency of the suppression strategy is significantly positively correlated with psychotic experiences. The observed pattern of habitual usage of emotion regulation strategies, such as greater use of suppression in individuals reporting greater psychotic experience, is in line with what has been found in previous studies [e.g., (51, 52)]. This suggests a maladaptive pattern of emotion regulation, in which individuals with greater distress from psychotic experiences repress the outward expression of their emotions rather than dealing with the emotional experience in a non-judgmental way. However, the inverse association between the use frequency of the reappraisal strategy and psychotic experiences was not confirmed in previous studies but in this study. This finding indicates that individuals who tend to use more often the reappraisal strategy report less distress from psychotic experiences. The individuals with the reappraisal pattern could change the cognitive meaning of the events, and therefore reduce the negative emotional responses (49, 53). This finding suggests that the reappraisal strategy could be a potential protective factor from psychotic experience.

We also confirmed the second hypothesis, that both strategies of emotion regulation mediated the relationship between personality traits and psychotic experiences. In the present study it was found that the reappraisal strategy significantly mediated the relationship between all five personality traits and psychotic experiences. The suppression strategy significantly mediated the relationship between such personality traits as neuroticism, extraversion, openness and agreeableness, and psychotic experiences. The construction of the personality traits manifests in groups of behavioral, emotional, and cognitive responses. The individuals with different personality traits (i.e., neuroticism and extraversion) employ emotion regulation strategies differently, which in turn influences the experiences of their emotions (54). These findings suggest that individuals with such personality traits as extraversion, conscientiousness, agreeableness and openness tend to use the reappraisal strategy, which may help to relieve the distress of psychotic experiences. On another hand, individuals with extraversion, openness, and agreeableness tend to reduce the use of the suppression strategy, which could also help to decrease the distress of psychotic experience, while individuals with neuroticism tend to use the suppression strategy and hence increase the distress of psychotic experience.

The current findings in this study suggest that certain emotion regulation styles and personality traits can function as protective factors against the psychotic experiences. This is particularly significant for suggesting interventions for non-clinical individuals with psychotic experiences. Although these experiences are distressing, they alone are not sufficient to warrant the use of anti-psychotic medication (55). Therefore, it is important to identify emotional and cognitive impact factors on psychotic experiences, which could function as potential targets of psychosocial intervention. The findings of the present study indicate that psychotherapy approaches that target improvement of emotion regulation skills could be particularly effective. Reappraisal was especially related to less distress from psychotic experiences, and it could be a potentially significant early intervention target in non-clinical individuals.

### Implication and Limitation

The results of this study should be interpreted in the context of several limitations. First, this study was implemented in a sample of college students, and to some extent it limits the feasibility of generalizing the conclusions. Second, we assessed distress of psychotic experiences via a self-reported instrument. It may lead to an overestimation of the prevalence of psychotic experiences due to the possible misinterpretation of the questions. Finally, the cross-sectional design of this study does not warrant causal inferences about the direction of the relationship between personality traits, emotion regulation and psychotic experiences. Whether adaptive emotion regulation can decrease the distress of psychotic experiences or, conversely, distress from psychotic experiences may promote maladaptive patterns of emotion regulation strategy use, needs to be further examined. Future research should seek to figure out these possibilities through a longitudinal, cross-lagged design.

Despite these limitations, the present findings emphasize the importance of personality traits and emotion regulation strategies for the mental health of youth in the general population and provide evidence connecting emotion regulation to the personality traits and psychotic experiences in the general population. These results suggest emotion regulation strategies as an important target for early intervention of psychotic and other psychiatric disorders.

## Ethics Statement

This study was approved by the Ethics Committee of Medicine and Life Science, Tongji University (Protocol no. 2016YXY07), with written informed consent from participants of the study.

## Author Contributions

JS contributed to study design, recruitment of participants, data analysis, and interpretation and writing of the manuscript. YY contributed to recruitment of participants. CZ and ZM contributed to recruitment of participants and interpretation of results. FY contributed to recruitment of participants. XZ contributed to study design and interpretation of results. All authors have approved the final manuscript.

### Conflict of Interest Statement

The authors declare that the research was conducted in the absence of any commercial or financial relationships that could be construed as a potential conflict of interest.
